# Effects of n-3 Long-Chain Polyunsaturated Fatty Acid and Vitamin D Supplementation on Transcriptional Profiles of Human Lung Organoids

**DOI:** 10.3390/metabo15100670

**Published:** 2025-10-14

**Authors:** Mina Ali, Martin Steen Mortensen, Ole Bæk, Nicklas Brustad, Tingting Wang, Liang Chen, Min Kim, Casper-Emil Tingskov Pedersen, Trevor D. Lawley, Athanasios Pasias, Jakub Sedzinski, Jakob Stokholm, Klaus Bønnelykke, Bo Chawes

**Affiliations:** 1 Copenhagen Prospective Studies on Asthma in Childhood (COPSAC), Herlev and Gentofte Hospital, University of Copenhagen, DK-2820 Gentofte, Denmark; mina.ali@dbac.dk (M.A.); ole.baek@sund.ku.dk (O.B.); nicklas.brustad@dbac.dk (N.B.); ting.wang@dbac.dk (T.W.); liang.chen@dbac.dk (L.C.); min.kim@dbac.dk (M.K.); casper.pedersen@dbac.dk (C.-E.T.P.); stokholm@copsac.com (J.S.); kb@copsac.com (K.B.); 2 National Food Institute, Technical University of Denmark, DK-2800 Kongens Lyngby, Denmark; masmo@food.dtu.dk; 3 Section for Comparative Pediatrics and Nutrition, Department of Veterinary and Animal Sciences, University of Copenhagen, DK-1870 Frederiksberg, Denmark; 4 BRIDGE Translational Excellence Programme, Faculty of Health and Medical Sciences, University of Copenhagen, DK-2200 Copenhagen, Denmark; 5 Host-Microbiota Interactions Laboratory, Wellcome Sanger Institute, Hinxton, Cambridge CB10 1SA, UK; tl2@sanger.ac.uk; 6 Novo Nordisk Foundation Center for Stem Cell Medicine (reNEW), Department of Biomedical Sciences, Faculty of Health and Medical Sciences, University of Copenhagen, DK-2200 Copenhagen, Denmark; pasias@sund.ku.dk (A.P.); jakub.sedzinski@sund.ku.dk (J.S.); 7 Section of Microbiology and Fermentation, Department of Food Science, University of Copenhagen, DK-1958 Frederiksberg, Denmark; 8 Department of Pediatrics, Slagelse Hospital, DK-4200 Slagelse, Denmark; 9 Department of Clinical Medicine, Faculty of Health and Medical Sciences, University of Copenhagen, DK-2200 Copenhagen, Denmark

**Keywords:** human lung organoid, asthma, n-3 long-chain polyunsaturated fatty acid, vitamin D, *CPT1A*, *ANGPTL4*, *CYP24A1*, PPAR signaling pathway

## Abstract

**Background/Objectives:** Randomized clinical trials (RCTs) suggest that n-3 long-chain polyunsaturated fatty acids (n-3 LCPUFA) and high-dose vitamin D supplementation during pregnancy may protect against childhood asthma. However, the underlying mechanisms remain unclear. **Methods:** To explore the transcriptional effects of various concentrations of n-3 LCPUFA and vitamin D supplementation on in utero lung development, we cultured human lung organoids derived from BILX and SEHP human-induced pluripotent stem cell lines at the Sanger Institute (Cambridge, UK). The organoids were treated with either no supplementation, or low (0.01 µL/mL) or high (0.1 µL/mL) concentrations of n-3 LCPUFA, as well as no supplementation, or low (5 pM) or high (50 pM) concentrations of vitamin D. Organoids were matured for 50 days, with foregut spheroids embedded in Matrigel and later re-embedded individually to ensure robust growth. We then assessed the impact of these supplementations using RNA sequencing. **Results:** RNA sequencing of four replicates per condition (36 total samples) revealed that n-3 LCPUFA supplementation had a more substantial impact on gene regulation than vitamin D (differentially expressed genes, *n* = 907 vs. *n* = 23). *CPT1A* and *ANGPTL4* genes were highly expressed in media cultured with a high concentration of n-3 LCPUFA, while *CYP24A1* was among the highly expressed genes in media cultured with a high concentration of vitamin D. Enrichment analysis showed activation of PPAR pathways, suggesting that n-3 LCPUFA supplementation may protect against asthma by regulating lipid metabolism and inflammation. **Conclusions:** We identified several genes and pathways that may provide insights into the biological effects of n-3 LCPUFA and vitamin D supplementation on asthma pathophysiology.

## 1. Introduction

Respiratory disorders, including asthma, represent a significant global health burden, affecting quality of life and imposing substantial healthcare costs [1,2]. Asthma alone affects 1–10% of children and adolescents in different countries [3]. Understanding the underlying mechanisms of respiratory diseases is crucial for developing effective preventive strategies and treatments.

Randomized clinical trials (RCTs), including those conducted by the Copenhagen Prospective Studies on Asthma in Childhood (COPSAC), have shown that supplementation during pregnancy with n-3 long-chain polyunsaturated fatty acids (n-3 LCPUFAs) and high-dose vitamin D can reduce the risk of asthma in the offspring [4,5,6]. Adequate intake of about 250–500 mg/day of n-3 LCPUFAs [7,8] and 600–800 IU/day of vitamin D [9] is recommended to achieve health benefits. However, due to the intricate architecture and cellular complexity of the developing human lung in utero, the underlying mechanisms remain poorly understood.

Advances in biomedical research have brought forward innovative in vitro 3D models, such as human lung organoids [10], which can dramatically enhance our ability to study respiratory diseases in a controlled yet more physiologically relevant environment [11,12]. This model system, derived from human pluripotent stem cells (hPSCs), embryonic stem cells (ESCs) and adult stem cells (ASCs), enables researchers to dissect specific cellular responses and molecular pathways activated in response to various treatments or conditions [13].

In this study, we aimed to investigate the transcriptional effect of n-3 LCPUFA and vitamin D supplementation in various concentrations on the development of human lung organoids. Building on the series of COPSAC intervention studies [4,14,15], we have demonstrated that maternal supplementation leads to measurable changes in circulating nutrient concentrations in both mothers and children. For the present organoid study, we therefore aimed to recapitulate physiologically relevant exposures in vitro. The concentrations of n-3 LCPUFAs were based on maternal plasma levels of EPA+DHA measured in the intervention and placebo arms at week 1 postpartum, thereby defining low and high concentrations representative of biologically relevant exposures. For vitamin D, we selected concentrations identical to those previously applied in human bronchial epithelial cell models, where they effectively modulated gene expression [16,17]. To extend the dose–response evaluation, we also included a no-supplementation control and a higher exposure level, ensuring that our experimental setup captured both physiological and supraphysiological ranges of nutrient availability.

**Figure 1:**
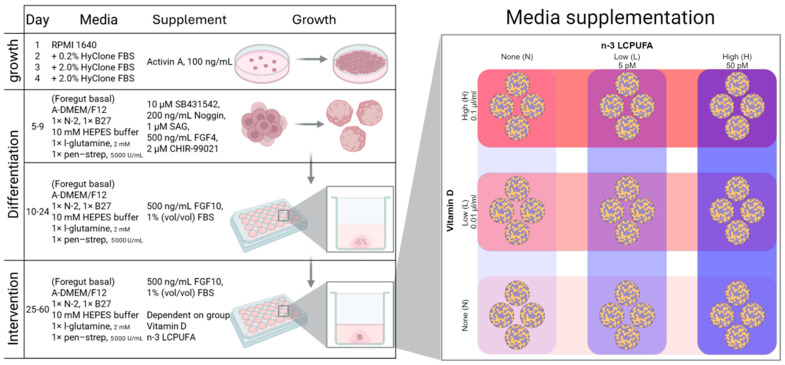
Experimental setup. Human lung organoids were generated twice, from BILX_2 and SEHP_2 cell lines. First foregut spheroids were generated, then harvested and 2–5 foregut spheroids embedded together in Matrigel drops. After 14 days the foregut spheroids were extracted and re-embedded individually in Matrigel drops. For the remaining 36 days of human lung organoid maturation, vitamin D and n-3 LCPUFA were added in a crossover design with none, low, and high levels of each and 4 replicates in each of the nine conditions. Upon full maturation, RNA was extracted from 4 replicates from each condition and RNA sequenced.

At Sanger institute (Cambridge, UK), we grew human lung organoids derived from the BILX_2 and SEHP_2 human-induced pluripotent stem cell (iPSC) lines, with none, low (0.01 µL/mL) and high (0.1 µL/mL) concentrations of n-3 LCPUFA and none, low (5 pM) and high (50 pM) concentrations of vitamin D in the media during maturation and measured the impact of the supplementations using RNA sequencing. The objective was to identify genes and biochemical pathways affected by the supplements, which could be important for asthma pathophysiology. An overview of the study is shown in (Figure 1).

## 2. Materials and Methods

### 2.1. Study Design

To investigate the transcriptional effects of n-3 LCPUFA and vitamin D on lung development, human lung organoids were cultured at the Sanger Institute (Cambridge, UK). These organoids were derived from the BILX_2 and SEHP_2 human iPSC lines, which had a normal disease status and were exposed to varying concentrations of n-3 LCPUFA (none, low at 0.03 µL/mL, and high at 0.3 µL/mL, DHA/EPA ratio: 1:2) and vitamin D (none, low at 5 pM, and high at 50 pM) in the media during maturation. The impact of these supplements was assessed using quantitative PCR (qPCR) and RNA sequencing. For each cell line, nine different media formulations were prepared based on the varying concentrations of n-3 LCPUFA and vitamin D. Each formulation was replicated four times, resulting in 36 distinct samples. However, one replicate from the media with a high concentration of n-3 LCPUFA and no vitamin D in BILX_2 was excluded due to poor quality.

### 2.2. Generation of Human iPSC-Derived Lung Organoids

Human lung organoids were generated following Miller et al., 2019 [18], with minor modifications (Figure 1). Briefly, iPSCs were directed to definitive endoderm for 4 days in RPMI 1640 medium with 100 ng/mL Activin A and 0–2% HyClone FBS. The cells were then patterned for 6 days toward anterior foregut endoderm in foregut basal media (Advanced DMEM/F12, 1× N-2 supplement, 1× B27 supplement, 10 mM HEPES buffer, 2 mM L-glutamine, and 5000 U/mL penicillin–streptomycin) with 10 µM SB431542, 200 ng/mL NOGGIN, 1 µM Smoothened agonist (SAG), 500 ng/mL FGF4 (recombinant human fibroblast growth factor 4), and 2 µM CHIR-99021. Cell morphology and growth were monitored by phase-contrast microscopy (EVOS XL Core, Thermo Fisher Scientific, Waltham, MA, USA). By day 10, self-assembled foregut spheroids were collected, embedded into Matrigel droplets (2–5 spheroids per droplet) and cultured in foregut basal medium supplemented with 1% FBS and FGF10 (500 ng/mL) to promote lung lineage specification and maturation. Cultures were maintained for 50 days with medium exchanges twice weekly and re-embedded into fresh Matrigel every 14 days; at the first re-embedding, spheroids were separated into individual droplets (one droplet per well of a 24-well plate) to support uniform growth. From the first re-embedding onward, the medium was supplemented with vitamin D (1α,25-dihydroxyvitamin D3, D5130, Sigma-Aldrich, St. Louis, MO, USA) and n-3 LCPUFA (cis-4,7,10,13,16,19-docosahexaenoic acid (DHA), D2534, Sigma-Aldrich, St. Louis, MO, USA; and cis-5,8,11,14,17-eicosapentaenoic acid (EPA), E2011, Sigma-Aldrich, St. Louis, MO, USA) at the indicated concentrations. EPA and DHA were supplied as liquids in sealed ampules and handled aseptically in a Class II biosafety cabinet without further sterilization. Vitamin D was dissolved in 95% ethanol to prepare 10 µM stock solutions, sterile-filtered (0.22 µm), aliquoted, and stored at −20 °C, protected from light. Working stocks (0.5 µM in ADMEM) were prepared immediately before use and diluted into culture medium to the final concentrations used in the experiments; the final ethanol content in culture was ≤0.01% (*v*/*v*). All additions were performed with sterile, single-use plasticware; UV sterilization was not employed. Organoids were recovered from Matrigel using Corning Cell Recovery Solution (354253, Corning, Wiesbaden, Germany) prior to downstream analyses. For each condition, 8–10 organoids were cultured to enable quality control (exclusion of aberrant structures) and to ensure sufficient material for analysis. When implementing the protocol in our laboratory, lineage fidelity and differentiation toward appropriate lung cell types were confirmed by qPCR of lineage-specific markers. The overall staging and timing (definitive endoderm → anterior foregut endoderm → foregut spheroids → long-term maturation with FGF10) mirror the progression described by Miller et al. for human lung organoid generation.

### 2.3. RNA Extraction and Gene Expression Profiling

RNA was extracted from four organoids from each condition, 36 organoids per cell line, using the RNeasy Mini Kit (Cat. No. 74106, Qiagen, Hilden, Germany) following manufacturer’s instructions and submitted to the DNA Pipeline Operations, Wellcome Sanger Institute, for library preparation and sequencing. The samples were sequenced 2 × 75 bp paired end on Illumina HiSeq 4000, with 7–8 samples per sequencing lane (72 samples and 2 negative controls on 10 lanes).

### 2.4. Bioinformatics Pipeline for Analyzing RNA-Seq Data

Initially, raw sequencing reads were quality-checked by assessing the base quality score distributions, sequence duplication levels, and overrepresented sequences using FastQC (v0.12.0) [19]. Next, Trimmomatic (v0.39) [20] was employed to trim adapter sequences and remove low-quality bases with a quality score threshold of 20. Cleaned reads were then aligned to the reference transcriptome to quantify the expression levels of transcripts using Salmon (v1.10.2) [21]. The output obtained from Salmon was a matrix of transcript abundance estimates, reported as counts and normalized counts (such as TPM—Transcripts Per Million, and effective counts which adjust for the effective length of the transcripts), and it was input into DESeq2 package (v1.44.0) [22] within the R statistical computing environment for differential expression analysis.

Within DESeq2, counts were first normalized to account for differences in sequencing depth and RNA composition across samples. Gene-specific dispersion estimates were then calculated to model overdispersion in the data. A generalized linear model (GLM) was fitted to the normalized counts, using the concentration of n-3 LCPUFA and vitamin D (none, low, and high) as the design factor. Each condition was represented by four technical replicates. Prior to differential expression testing, lowly expressed genes were filtered out by retaining only those with a total count ≥10 across all samples and counts ≥10 in at least two technical replicates per group. Differential expression between conditions was assessed using Wald tests or likelihood ratio tests, and *p*-values were adjusted for multiple testing with the Benjamini–Hochberg (BH) procedure. Genes with an adjusted *p*-value < 0.05 and an absolute log2 fold change ≥ 1 were considered differentially expressed. MA plots and volcano plots were generated to visualize the magnitude of expression changes and statistical significance.

Principal Coordinates Analysis (PCoA) was performed to evaluate sample clustering and identify potential outliers. To statistically compare similarities between different media, permutational multivariate analysis of variance (PERMANOVA) was conducted using the adonis function from the *vegan* package in R (version 4.5.0) [23], and *p*-values were adjusted using the BH method.

To identify significant biological pathways and processes, we performed pathway enrichment analysis using the pathfinder [24] package in R, with the Kyoto Encyclopedia of Genes and Genomes (KEGG) database. The input consisted of significantly differentially expressed genes (adjusted *p*-value < 0.05), and enrichment significance was determined using the false discovery rate (FDR) method, with pathways having FDR < 0.05 considered significantly enriched.

## 3. Results

### 3.1. Quality Assessment of Lung Organoid Cell Cultures

As part of implementing the human lung organoid derivation protocol [18], the presence of lung specific cell type markers was verified using qPCR with foregut spheroids as controls. We confirmed the presence of basal stem-cell-like cell marker (P63), ciliated cell marker (FOXj1), alveolar type I cell markers (HOPX, SOX9), alveolar type II cell marker (SFTPC), goblet cell marker (MUC5AC). Finally, the successful growth of the human lung organoids was assessed visually (Supplementary Figure S1).

### 3.2. Quality Assessment of Transcriptome Data

To assess the overall gene expression levels across samples and to detect outliers, we generated boxplots of Transcripts Per Million (TPM)-normalized read counts on a log scale for each sample and plotted their density distributions (Supplementary Figure S2A,B). All samples exhibited tight clustering of boxplot elements (medians, whiskers, and fences) and followed similar distributions, indicating good experimental consistency. The density plots revealed two peaks, suggesting the presence of two distinct groups of genes with high and low expression profiles. The peak at a TPM of 0.1 (log scale) indicates that many genes were either not expressed or were expressed at very low levels, while the second peak around TPM 2500 represents a group of genes with substantially higher expression (Supplementary Figure S2B). In Principal Coordinates Analysis (PCoA) plots, some replicates diverged, potentially due to genetic differences or the biological complexity of the samples (Supplementary Figure S2C).

### 3.3. Differential Expression Analysis of BILX_2 Cell Line Across Various Media Conditions

**Figure 2:**
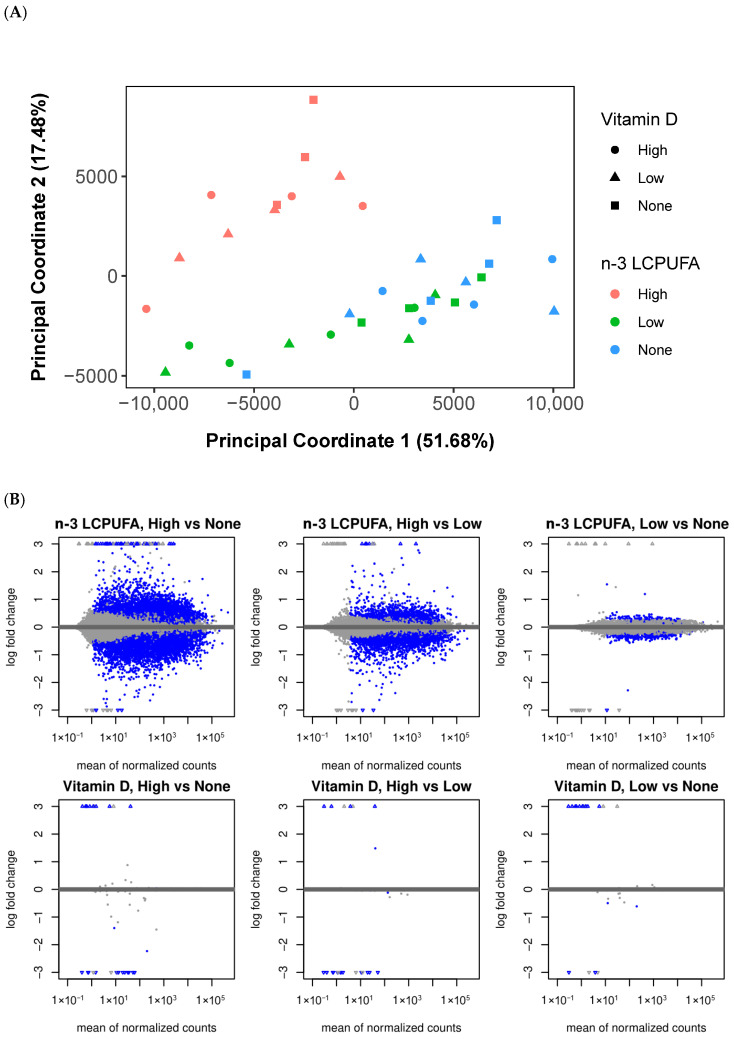

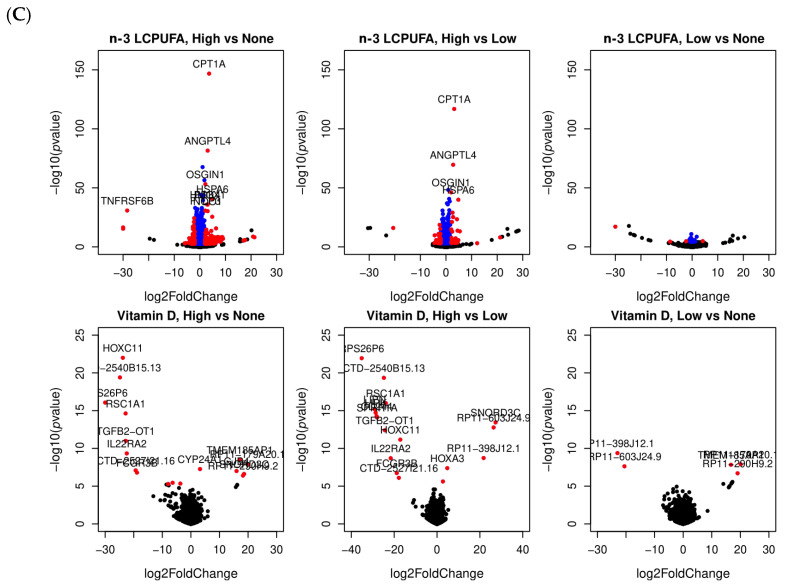
Differential expression analysis between different concentrations of n-3 LCPUFA and vitamin D. (**A**) Principal Coordinates Analysis (PCoA) plot of replicates: Each dot represents a sample, with dot color indicating the concentration of n-3 LCPUFA and dot shape indicating the concentration of Vitamin D. (**B**) MA plot of shrunken Log2 fold changes over mean of normalized counts: Points are colored blue if the Benjamini–Hochberg (BH) adjusted *p*-value is less than 0.05. Points with a log2 fold change greater than 3 are represented as open triangles pointing up or down, indicating substantial expression changes. (**C**) Volcano plot showing statistical significance and magnitude of change for genes: The X-axis represents the log2 fold change, indicating the magnitude of gene expression change, while the Y-axis represents the statistical significance (−log10 *p*-value). Points are blue if the BH adjusted *p*-value is <0.01, and red if log2 fold change > 1 and BH adjusted *p*-value < 0.01. Gene labels are shown for a subset of the genes.

To evaluate the similarities and differences among various media conditions, we generated PCoA plots of the samples and observed that media containing a high concentration of n-3 LCPUFA formed a distinct cluster, separate from other media (Figure 2A). There was no clear clustering based on vitamin D, as media with varying vitamin D concentrations were scattered throughout the PCoA plot (Figure 2A). The PERMANOVA analysis indicated that n-3 LCPUFA concentration significantly influenced the variation in gene expression profiles (*p* = 0.001), whereas vitamin D levels did not have a statistically significant effect. Post hoc pairwise tests further confirmed that media containing a high concentration of n-3 LCPUFA had significantly different gene expression profiles compared to those with low or no n-3 LCPUFA (BH adjusted *p* = 0.003).

To visualize the overall distribution of changes in gene expression between different media, we generated MA plots of the shrunken log2 fold changes over the mean of normalized counts for each gene, highlighting differentially expressed genes with a BH adjusted *p*-value less than 0.05 (Figure 2B). In general, more genes were affected by n-3 LCPUFA than by vitamin D. Specifically, media containing a high concentration of n-3 LCPUFA had the highest number of differentially expressed genes compared to media without n-3 LCPUFA. The list of differentially expressed genes with a BH adjusted *p*-value less than 0.05 and at least a 2-fold change is shown in Supplementary Table S1A–F.

A total of 907 genes were differentially expressed in media with a high concentration of n-3 LCPUFA compared to media without any n-3 LCPUFA added. In contrast, only 4 genes were differentially expressed in media with a low concentration of n-3 LCPUFA compared to none, and 168 genes were differentially expressed when comparing high- versus low-concentration n-3 LCPUFA (Supplementary Table S1A–C).

For vitamin D, only 23 genes were differentially expressed in media with a high concentration of vitamin D compared to media without any vitamin D added. A total of 17 genes were differentially expressed when comparing high and low concentrations of vitamin D, and 13 genes were differentially expressed when comparing low concentration to no vitamin D (Supplementary Table S1D–F).

The *CPT1A* (Carnitine Palmitoyltransferase 1A) and *ANGPTL4* (Angiopoietin-Like 4) genes, upregulated in media with high concentrations of n-3 LCPUFA, were the top hits in the differential expression analysis. When comparing high versus no concentration, *CPT1A* showed a log2 fold change of 3.6 (BH-*p* = 4.33 × 10^−143^) and *ANGPTL4* a log2 fold change of 3.02 (BH-*p* = 3.76 × 10^−78^). In the comparison of high versus low concentration, *CPT1A* exhibited a log2 fold change of 3.19 (BH-*p* = 3.16 × 10^−113^) and *ANGPTL4* a log2 fold change of 2.77 (BH-*p* = 2.85 × 10^−66^) (Figure 2C and Supplementary Table S1A,B).

Among the top hits in differential expression analysis between media with high- concentration vitamin D and media with no or low concentrations, the *CYP24A1* (Cytochrome P450 Family 24 Subfamily A Member 1) gene (log2 fold change = 3.14, BH-*p* = 1.7 × 10^−4^) is directly related to vitamin D metabolism (Figure 2C and Supplementary Table S1D,E).

### 3.4. Combined Effect of n-3 LCPUFA and Vitamin D in Different Media on BILX_2 Cell Line

To investigate the combined effects of n-3 LCPUFA and vitamin D and to identify genes significantly influenced by varying concentrations of these compounds, we performed differential gene expression analysis on media with different combinations of n-3 LCPUFA and vitamin D. Consistent with our previous analysis, in media with high concentrations of n-3 LCPUFA, we observed the highest number of differentially expressed genes compared with low and no concentration of n-3 LCPUFA and the *CPT1A* and *ANGPTL4* genes were among the most highly expressed genes (Supplementary Figure S3A,B and Table S2A–I).

The highest number of differentially expressed genes was observed in the comparison between high versus no concentration of n-3 LCPUFA in media containing a low concentration of vitamin D, with 591 genes showing at least a 2-fold change and a BH-adjusted *p*-value of less than 0.05 (Supplementary Table S2B). Among the differentially expressed genes, we identified *CCL11* (C-C Motif Chemokine Ligand 11), which is known for its role in attracting eosinophils that is involved in allergic reactions and parasitic infections [25].

In media without vitamin D, *CCL11* expression was significantly lower under high n-3 LCPUFA concentration compared with both no n-3 LCPUFA (log2 fold change: −21.51, BH-*p* = 1.58 × 10^−6^) and low n-3 LCPUFA (log2; fold change: −5.72, BH-*p* = 0.002) (Supplementary Table S2A–D). However, in media with a low concentration of vitamin D, *CCL11* expression was significantly higher at high n-3 LCPUFA concentration compared to no n-3 LCPUFA (log2 fold change: 19.00, BH-*p* < 0.001) (Supplementary Figure S3B and Table S2B) and at low n-3 LCPUFA concentration compared to no concentration (log2 fold change: 20.22, BH-*p* = 5.98 × 10^−6^) (Supplementary Figure S3B and Table S2H). Additionally, in media with a high concentration of n-3 LCPUFA, *CCL11* expression was significantly higher in media containing a high concentration of vitamin D compared to no concentration (log2 fold change: 18.90, BH-*p* = 10^−4^) (Supplementary Figure S4B and Table S3C). As such, it seems that induction of *CCL11* by n-3 LCPUFA increases with increasing Vitamin D levels.

### 3.5. Gene Set Enrichment and Pathway Analysis of n-3 LCPUFA

**Figure 3:**
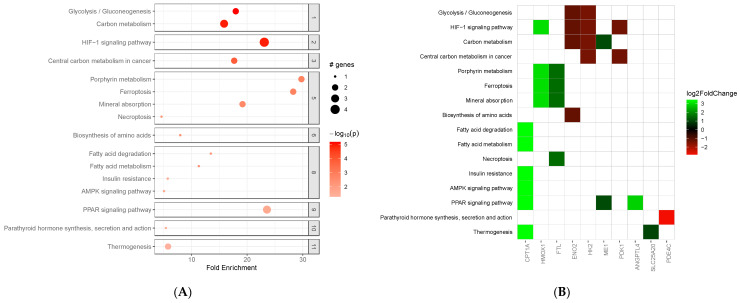
Key enriched KEGG pathways involving genes differentially expressed in media with a high concentration of n-3 LCPUFA. (**A**) Enrichment plot displaying fold enrichment values, adjusted −log10 (*p*-value), and the number of genes for each enriched pathway; (**B**) Heatmap showing log2 fold changes of genes within enriched pathways.

A total of 56 genes were consistently differentially expressed when comparing high versus no concentration of n-3 LCPUFA, independent of vitamin D concentration. We conducted a gene set enrichment analysis using the KEGG pathway library to identify the biological pathways associated with these genes. Figure 3A,B and Supplementary Table S4 highlights the key enriched pathways involving these genes. *CPT1A* and *ANGPTL4* are both involved in the PPAR (Peroxisome Proliferator-Activated Receptor) signaling pathway, a nuclear hormone receptor activated by fatty acids and playing critical roles in lung development, tissue repair, and immune responses [26,27]. Furthermore, *CPT1A* is implicated in other pathways, including fatty acid degradation, fatty acid metabolism, and the AMPK (AMP-activated protein kinase) signaling pathway. The AMPK pathway has anti-inflammatory effects and protects lung epithelial cells from oxidative stress and inflammation [28].

### 3.6. Effect of n-3 LCPUFA and Vitamin D on SEHP_2 Induced Pluripotent Stem Cell Line

We repeated the same procedures using the SEHP_2 human-induced pluripotent stem cell line. In the quality assessment, TPM boxplots of the samples exhibited tight clustering, and the density plot showed two peaks at low and high TPM levels. In the PCoA plots, we observed distance between replicates (Supplementary Figure S5A–C). Unlike the BILX_2 cell line, there was no distinct cluster for media containing a high concentration of n-3 LCPUFA (Supplementary Figure S5D).

In SEHP_2, fewer genes were differentially expressed compared to the BILX_2 cell line. Like BILX_2, the largest number of differentially expressed genes was observed when comparing high versus no concentrations of n-3 LCPUFA, with 53 genes showing differential expression. Only 6 genes were differentially expressed in the comparison of media with a low concentration and no concentration of n-3 LCPUFA, and 40 genes were differentially expressed when comparing high to low concentrations of n-3 LCPUFA. Supplementary Table S5A–F display the list of differentially expressed genes with a BH adjusted *p*-value of less than 0.05 and a minimum of a 2-fold change.

Notably, the *CPT1A* and *ANGPTL4* genes were again among the most significantly upregulated genes in media with a high-concentration of n-3 LCPUFA (Supplementary Figure S6B). When we compared the lists of differentially expressed genes for both cell lines in the high- concentration versus no concentration of n-3 LCPUFA comparison, we found 16 common differentially expressed genes. These genes included *CPT1A*, *ANGPTL4*, *PDK4*, *HAS1*, *SLC16A6*, *HMGCS2*, *CLDN10*, *ADM*, *OLAH*, *NMRAL2P*, *NQO1*, *NYX*, *HAR1A*, *AC002451.3*, *RP11-737O24.2*, and *SPEM3*. A pathway enrichment analysis showed that these genes are involved in the PPAR signaling pathway (*CPT1A*, *ANGPTL4*, and *HMGCS2*), fatty acid metabolism (*CPT1A* and *HMGCS2*), xenobiotic metabolism (*PDK4* and *NQO1*) and KRAS signaling (*ANGPTL4*). Supplementary Table S8 displays the fold-change ratios and BH-adjusted *p*-values in both cell lines.

For vitamin D, we did not identify any common differentially expressed genes between the two cell lines when comparing high and low concentrations of vitamin D to none.

## 4. Discussion

In this study, we utilized human-derived lung organoid cultures to investigate transcriptional responses to n-3 LCPUFA and vitamin D, with an emphasis on how these nutrients influence gene expression profiles relevant to healthy lung development and asthma-related pathophysiology. Our gene expression analysis revealed that a greater number of genes were significantly regulated by n-3 LCPUFA compared with vitamin D supplementation, suggesting that n-3 LCPUFAs may influence more extensive molecular networks involved in lung development in utero that could influence asthma susceptibility, while the effects of vitamin D intervention may be on other target organs than the lungs. This is in line with the proven diverse roles of n-3 LCPUFAs across various biological processes, including lipid metabolism [29], inflammation [30,31] and cellular function. The n-3 LCPUFAs regulate lipid metabolism by influencing pathways involved in lipid synthesis and breakdown, thereby contributing to cellular energy balance and membrane structure. In inflammation, n-3 LCPUFAs play an anti-inflammatory role through the production of specialized pro-resolving mediators such as resolvins and protectins, which actively resolve inflammation [31]. Additionally, n-3 LCPUFAs modulate transcription factors such as PPARs that we observed in our gene enrichment analyses, which are pivotal in regulating gene expression related to inflammation, lipid metabolism, and cellular differentiation [32]. When activated by n-3 LCPUFAs, PPARs regulate genes involved in lipid metabolism, inflammation reduction, and glucose homeostasis, contributing to anti-inflammatory and lipid-lowering effects. Activation of PPARγ has been shown to downregulate pro-inflammatory cytokines and decrease airway hyperresponsiveness, a hallmark of asthma [33]. The PPARγ agonist rosiglitazone has been shown to decrease airway inflammation and remodeling in murine models of asthma by activating the PPARγ/HO-1 signaling pathway [34]. Furthermore, studies have demonstrated that PPARγ activation enhances the expression of glucocorticoid-induced leucine zipper (GILZ), an anti-inflammatory protein, thereby amplifying its role in suppressing inflammatory responses in asthma [35,36].

The *CPT1A* and *ANGPTL4* genes, both significantly upregulated under high concentration of n-3 LCPUFA in our experiment, play important roles in the PPAR pathway, particularly in lipid metabolism and anti-inflammatory responses. *CPT1A* encodes an enzyme responsible for transporting long-chain fatty acids into mitochondria, a critical step for fatty acid β-oxidation and energy production, particularly when glucose availability is limited [37]. *ANGPTL4* encodes a protein that regulates lipid metabolism and contributes to inflammation control and energy homeostasis [38].

PPARα activation promotes *CPT1A* expression, enhancing fatty acid breakdown and energy production, which supports the energy demands of lung function [39]. *ANGPTL4*, a PPARγ target gene, modulates lipid metabolism by inhibiting lipoprotein lipase (LPL), regulating lipid storage and mobilization [40]. These roles collectively highlight the broad and critical functions of n-3 LCPUFAs in maintaining homeostasis, which may be important for preventing chronic lung diseases such as childhood asthma.

Vitamin D functions through various pathways through binding to the vitamin D receptor (VDR) and its direct effects are in multiple areas such as bone health [41], calcium metabolism [42], infection risk [43], immune function [44,45] and asthma [6] among others. It facilitates the absorption of calcium and phosphate, maintaining adequate serum levels of these minerals essential for normal bone mineralization. In the immune system, vitamin D plays a crucial role in modulating both innate and adaptive responses. The active form, 1,25-dihydroxyvitamin D3, interacts with the intracellular VDR expressed in immune cells such as monocytes, macrophages, dendritic cells, and T and B lymphocytes, enhancing their pathogen-fighting capabilities [46,47]. Additionally, vitamin D reduces the release of pro-inflammatory cytokines while enhancing the expression of anti-inflammatory cytokines, helping to regulate inflammation and immune tolerance [46]. In our experiment, *CYP24A1*, which is responsible for the catabolism of the active form of vitamin D, was upregulated following high-dose vitamin D supplementation. This gene encodes an enzyme that degrades active vitamin D metabolites, helping to maintain the balance of vitamin D and calcium in the body [48]. Both genetic variants in VDR and *CYP24A1* have been associated with risk of asthma and atopy [49].

It is important to acknowledge that while both n-3 LCPUFAs and vitamin D influence immune and inflammatory responses, their mechanisms of action are distinct and may have both synergistic and opposing effects on lung development in utero. An example of this is *CCL11*, which exhibited opposing effects in our stratified analysis. *CCL11* was downregulated in media with a high concentration of n-3 LCPUFA and no vitamin D, whereas it was upregulated in media with a high concentration of n-3 LCPUFA and low vitamin D. Elevated levels of *CCL11* were commonly observed in patients with asthma, allergic rhinitis, and atopic dermatitis [50]. Studies have shown that the *CCL11* rs2302009 (A>G) variant is linked to an increased risk of asthma, particularly in individuals carrying the G allele, which causes a threonine-to-alanine substitution at position 23 in the CCL11 protein. This substitution elevates plasma CCL11 levels, enhancing eosinophil recruitment to the airways, leading to increased allergic responses and intensified airway inflammation, which contribute to asthma susceptibility [51,52]. However, the biological significance of these transcriptional changes remains uncertain, and further validation is required to clarify their role in lung development and asthma susceptibility.

This study provides preliminary insights into the transcriptional effects of n-3 LCPUFA and vitamin D on lung development. However, linking these findings to asthma prevention in clinical settings requires several additional steps, including validation in future studies. The relationship between the observed gene expression changes and actual asthma outcomes is shaped by multiple layers of biological complexity; therefore, our results should be regarded as hypothesis-generating rather than directly predictive of clinical benefit.

In addition, several other limitations should be considered. Firstly, human iPSC-derived lung organoids, while replicating key structural and functional aspects of the human lung, primarily represent immature tissue and lack the full complexity of the lung microenvironment, including vasculature, immune cells, and mechanical forces such as airflow [12,53]. They may not fully capture the diversity of native lung cell types, including immune cells, nor accurately model chronic disease progression, long-term immune responses, or fibrosis, as they often represent early-stage tissue states [54]. As standalone systems, organoids are limited in their ability to mimic interactions between the lungs and other organs, such as the heart or immune system. A notable limitation of our study is that the effects of vitamin D on gene expression were less pronounced compared to those of n-3 LCPUFA. This discrepancy may not solely reflect a diminished biological role of vitamin D in lung development but could instead be attributed to specific limitations in our experimental setup. Human-derived iPSC lung organoids, while a powerful model for studying lung biology, may not fully recapitulate the cellular diversity and complexity of the developing lung. It is plausible that certain cell types or niches that are critical targets of vitamin D action were underrepresented or absent in our organoid cultures. For instance, vitamin D is known to exert regulatory effects on immune and mesenchymal cells, which might not be adequately represented in our lung organoid system. Additionally, vitamin D signaling could involve crosstalk with other cell types or systems not modeled in this in vitro setup.

Secondly, human iPSC-derived lung organoids encounter inherent challenges in reproducibility due to genetic variability between cell lines. Additionally, the differentiation protocols used to generate organoids vary across experiments and laboratories, resulting in inconsistencies. These challenges highlight the importance of developing complementary models that more accurately replicate in vivo conditions. Specifically, these limitations may have influenced our findings in the SEHP_2 human iPSC line. The substantial differences we observed between the BILX_2 and SEHP_2 cell lines, particularly in response to vitamin D, further underscore the challenges of reproducibility and generalizability in this model. Without replication in additional cell lines, it is difficult to determine whether these divergent responses reflect inherent genetic differences, epigenetic variation, or technical variability.

Thirdly, the specific concentrations of n-3 LCPUFA and vitamin D used in this study may not fully capture the range of physiological responses. This highlights the need for further research to assess dose–response relationships and the long-term effects of these supplements on lung development and disease prevention. While our concentrations were chosen to reflect biologically relevant exposures, it remains possible that different levels—or combinations of nutrients—could produce alternative effects. Moreover, in vitro systems cannot fully replicate the complex pharmacokinetics and metabolism of these nutrients in vivo, which may further influence their biological impact.

## 5. Conclusions

In this study, we explored the transcriptional effects of n-3 LCPUFA and vitamin D supplementation on human lung organoid cultures as a model of early lung development. Our findings demonstrate that n-3 LCPUFA exert broader and more pronounced effects on gene expression compared to vitamin D, particularly through the regulation of pathways related to lipid metabolism and inflammation. Notably, high concentration of n-3 LCPUFA upregulated *CPT1A* and *ANGPTL4*, key genes involved in the PPAR signaling pathway, underscoring potential molecular mechanisms by which n-3 LCPUFAs may influence asthma susceptibility.

These results provide preliminary insights into how nutritional factors may shape lung development at the molecular level. However, given the limitations of the organoid model and the exploratory nature of this work, the data should be interpreted as hypothesis-generating rather than directly predictive of clinical outcomes. Future studies, including replication in additional cell lines and in vivo validation, are needed to confirm the biological effects of n-3 LCPUFA and vitamin D in relation to lung development and asthma prevention.

## Data Availability

The original contributions presented in this study are included in the article/supplementary material. Further inquiries can be directed to the corresponding author.

## Supplementary Materials

The following supporting information can be downloaded at: https://www.mdpi.com/article/10.3390/metabo15100670/s1, Figure S1: Quality assessment of lung organoid cell cultures in different stages. Figure S2: Quality assessment of transcriptome data in the BILX_2 cell line. (A) Boxplots of TPM (Transcripts Per Million) normalized read counts on a log scale. The first character represents the n-3 LCPUFA concentration, and the second character represents the vitamin D concentration, with concentration designated as follows: N for none, L for low, and H for high. (B) Density plot of TPM normalized read counts. (C) Principal Coordinates Analysis (PCoA) plot of replicates in media with different concentrations of n-3 LCPUFA and vitamin D. The cell line name (BILX_2) is followed by two characters. The first character represents the n-3 LCPUFA concentration, and the second character represents the vitamin D concentration, with concentrations designated as follows: N for none, L for low, and H for high. Figure S3: Differential expression analysis between different concentrations of n-3 LCPUFA in media with varying concentrations of vitamin D. (A) MA plot of shrunken Log2 fold changes over the mean of normalized counts. Blue points indicate BH adjusted *p*-value < 0.05. Blue triangles (up/down) mark genes with log2 fold change > 3. (B) Volcano plot showing statistical significance and magnitude of change for genes. X-axis shows log2 fold change; Y-axis shows statistical significance (−log10 *p*-value). Blue points: BH adjusted *p*-value < 0.01; red points: log2 fold change > 1 and BH adjusted *p*-value < 0.01. Gene labels are shown for a subset of the genes. Figure S4: Differential expression analysis between different concentrations of vitamin D in media with varying concentrations of n-3 LCPUFA. (A) MA plot of shrunken Log2 fold changes over the mean of normalized counts. Blue points indicate BH adjusted *p*-value < 0.05. Blue triangles (up/down) mark genes with log2 fold change > 3. (B) Volcano plot showing statistical significance and magnitude of change for genes. X-axis shows log2 fold change; Y-axis shows statistical significance (−log10 *p*-value). Blue points: BH adjusted *p*-value < 0.01; red points: log2 fold change > 1 and BH adjusted *p*-value < 0.01. Gene labels are shown for a subset of the genes. Figure S5: Quality assessment of transcriptome data in the SEHP_2 cell line. (A) Boxplots of TPM (Transcripts Per Million) normalized read counts on a log scale. (B) Density plot of TPM normalized read counts. (C) Principal Coordinates Analysis (PCoA) plot of replicates in media with different concentrations of n-3 LCPUFA and vitamin D. The cell line name (SEHP_2) is followed by two characters. The first character represents the n-3 LCPUFA concentration, and the second character represents the vitamin D concentration, with concentrations designated as follows: N for none, L for low, and H for high. (D) PCoA plot of replicates: Each dot represents a sample, with dot color indicating the concentration of n-3 LCPUFA and dot shape indicating the concentration of Vitamin D. Figure S6: Differential expression analysis between different concentrations of n-3 LCPUFA and vitamin D in the SEHP_2 cell line. (A) MA plot of shrunken Log2 fold changes over mean of normalized counts: Points are colored blue if the Benjamini–Hochberg (BH) adjusted *p*-value is less than 0.05. Points with a log2 fold change greater than 3 are represented as open triangles pointing up or down, indicating substantial expression changes. (B) Volcano plot showing statistical significance and magnitude of change for genes. X-axis shows log2 fold change; Y-axis shows statistical significance (−log10 *p*-value). Blue points: BH adjusted *p*-value < 0.01; red points: log2 fold change > 1 and BH adjusted *p*-value < 0.01. Gene labels are shown for a subset of the genes. Figure S7: Differential expression analysis between different concentrations of n-3 LCPUFA in media with varying concentrations of vitamin D in the SEHP_2 cell line. (A) MA plot of shrunken Log2 fold changes over the mean of normalized counts. Blue points indicate BH adjusted *p*-value < 0.05. Blue triangles (up/down) mark genes with log2 fold change > 3. (B) Volcano plot showing statistical significance and magnitude of change for genes. X-axis shows log2 fold change; Y-axis shows statistical significance (−log10 *p*-value). Blue points: BH adjusted *p*-value < 0.01; red points: log2 fold change > 1 and BH adjusted *p*-value < 0.01. Gene labels are shown for a subset of the genes. Figure S8: Differential expression analysis between different concentrations of vitamin D in media with varying concentrations of n-3 LCPUFA in the SEHP_2 cell line. (A) MA plot of shrunken Log2 fold changes over the mean of normalized counts. Blue points indicate BH adjusted *p*-value < 0.05. Blue triangles (up/down) mark genes with log2 fold change > 3. (B) Volcano plot showing statistical significance and magnitude of change for genes. X-axis shows log2 fold change; Y-axis shows statistical significance (−log10 *p*-value). Blue points: BH adjusted *p*-value < 0.01; red points: log2 fold change > 1 and BH adjusted *p*-value < 0.01. Gene labels are shown for a subset of the genes. Table S1. Differential gene expression analysis under varying concentrations of n-3 LCPUFA and vitamin D. Genes with a Benjamini–Hochberg (BH) adjusted *p* value < 0.05 and an absolute log2 fold change ≥ 1 (corresponding to at least a 2-fold change) are shown. Sample names consist of the cell line identifier (BILX_2) followed by two characters indicating the treatment conditions: the first character represents the n-3 LCPUFA concentration, and the second character represents the vitamin D concentration, with N = none, L = low, and H = high. Expression values are reported as DESeq2-normalized read counts for all technical replicates. (A) Comparison between media with high versus no n-3 LCPUFA. (B) Comparison between media with high versus low n-3 LCPUFA. (C) Comparison between media with low versus no n-3 LCPUFA. (D) Comparison between media with high versus no vitamin D. (E) Comparison between media with high versus low vitamin D. (F) Comparison between media with low versus no vitamin D. Table S2. Differential gene expression analysis of the combined effects of n-3 LCPUFA and vitamin D. Genes with a Benjamini–Hochberg (BH) adjusted *p* value < 0.05 and an absolute log2 fold change ≥ 1 (corresponding to at least a 2-fold change) are shown. Sample names consist of the cell line identifier (BILX_2) followed by two characters indicating the treatment conditions: the first character represents the n-3 LCPUFA concentration, and the second character represents the vitamin D concentration, with N = none, L = low, and H = high. Expression values are reported as DESeq2-normalized read counts for all technical replicates. (A) Comparison between media with high versus no n-3 LCPUFA under conditions without vitamin D supplementation. (B) Comparison between media with high versus no n-3 LCPUFA under conditions with a low concentration of vitamin D. (C) Comparison between media with high versus no n-3 LCPUFA under conditions with a high concentration of vitamin D. (D) Comparison between media with high versus low n-3 LCPUFA under conditions without vitamin D supplementation. (E) Comparison between media with high versus low n-3 LCPUFA under conditions with a low concentration of vitamin D. (F) Comparison between media with high versus low n-3 LCPUFA under conditions with a high concentration of vitamin D. (G) Comparison between media with low versus no n-3 LCPUFA under conditions without vitamin D supplementation. (H) Comparison between media with low versus no n-3 LCPUFA under conditions with a low concentration of vitamin D. (I) Comparison between media with low versus no n-3 LCPUFA under conditions with a high concentration of vitamin D. Table S3. Differential gene expression analysis of the combined effects of vitamin D and n-3 LCPUFA. Genes with a Benjamini–Hochberg (BH) adjusted *p* value < 0.05 and an absolute log2 fold change ≥ 1 (corresponding to at least a 2-fold change) are shown. Sample names consist of the cell line identifier (BILX_2) followed by two characters indicating the treatment conditions: the first character represents the n-3 LCPUFA concentration, and the second character represents the vitamin D concentration, with N = none, L = low, and H = high. Expression values are reported as DESeq2-normalized read counts for all technical replicates. (A) Comparison between media with high versus no vitamin D under conditions without n-3 LCPUFA supplementation. (B) Comparison between media with high versus no vitamin D under conditions with a low concentration of n-3 LCPUFA. (C) Comparison between media with high versus no vitamin D under conditions with a high concentration of n-3 LCPUFA. (D) Comparison between media with high versus low vitamin D under conditions without n-3 LCPUFA supplementation. (E) Comparison between media with high versus low vitamin D under conditions with a low concentration of n-3 LCPUFA. (F) Comparison between media with high versus low vitamin D under conditions with a high concentration of n-3 LCPUFA. (G) Comparison between media with low versus no vitamin D under conditions without n-3 LCPUFA supplementation. (H) Comparison between media with low versus no vitamin D under conditions with a low concentration of n-3 LCPUFA. (I) Comparison between media with low versus no vitamin D under conditions with a high concentration of n-3 LCPUFA. Table S4. Key enriched KEGG pathways involving genes differentially expressed in media with a high concentration of n-3 LCPUFA. The table lists pathway terms, fold enrichment values, occurrence, support, and corresponding *p*-values. Genes significantly up- or down-regulated within each pathway are indicated in the respective columns. Table S5. Differential gene expression analysis under varying concentrations of n-3 LCPUFA and vitamin D. Genes with a Benjamini–Hochberg (BH) adjusted *p* value < 0.05 and an absolute log2 fold change ≥ 1 (corresponding to at least a 2-fold change) are shown. Sample names consist of the cell line identifier (SEHP_2) followed by two characters indicating the treatment conditions: the first character represents the n-3 LCPUFA concentration, and the second character represents the vitamin D concentration, with N = none, L = low, and H = high. Expression values are reported as DESeq2-normalized read counts for all technical replicates. (A) Comparison between media with high versus no n-3 LCPUFA. (B) Comparison between media with high versus low n-3 LCPUFA. (C) Comparison between media with low versus no n-3 LCPUFA. (D) Comparison between media with high versus no vitamin D. (E) Comparison between media with high versus low vitamin D. (F) Comparison between media with low versus no vitamin D. Table S6. Differential gene expression analysis of the combined effects of n-3 LCPUFA and vitamin D. Genes with a Benjamini–Hochberg (BH) adjusted *p* value < 0.05 and an absolute log2 fold change ≥ 1 (corresponding to at least a 2-fold change) are shown. Sample names consist of the cell line identifier (SEHP_2) followed by two characters indicating the treatment conditions: the first character represents the n-3 LCPUFA concentration, and the second character represents the vitamin D concentration, with N = none, L = low, and H = high. Expression values are reported as DESeq2-normalized read counts for all technical replicates. (A) Comparison between media with high versus no n-3 LCPUFA under conditions without vitamin D supplementation. (B) Comparison between media with high versus no n-3 LCPUFA under conditions with a low concentration of vitamin D. (C) Comparison between media with high versus no n-3 LCPUFA under conditions with a high concentration of vitamin D. (D) Comparison between media with high versus low n-3 LCPUFA under conditions without vitamin D supplementation. (E) Comparison between media with high versus low n-3 LCPUFA under conditions with a low concentration of vitamin D. (F) Comparison between media with high versus low n-3 LCPUFA under conditions with a high concentration of vitamin D. (G) Comparison between media with low versus no n-3 LCPUFA under conditions without vitamin D supplementation. (H) Comparison between media with low versus no n-3 LCPUFA under conditions with a low concentration of vitamin D. (I) Comparison between media with low versus no n-3 LCPUFA under conditions with a high concentration of vitamin D. Table S7. Differential gene expression analysis of the combined effects of vitamin D and n-3 LCPUFA. Genes with a Benjamini–Hochberg (BH) adjusted *p* value < 0.05 and an absolute log2 fold change ≥ 1 (corresponding to at least a 2-fold change) are shown. Sample names consist of the cell line identifier (SEHP_2) followed by two characters indicating the treatment conditions: the first character represents the n-3 LCPUFA concentration, and the second character represents the vitamin D concentration, with N = none, L = low, and H = high. Expression values are reported as DESeq2-normalized read counts for all technical replicates. (A) Comparison between media with high versus no vitamin D under conditions without n-3 LCPUFA supplementation. (B) Comparison between media with high versus no vitamin D under conditions with a low concentration of n-3 LCPUFA. (C) Comparison between media with high versus no vitamin D under conditions with a high concentration of n-3 LCPUFA. (D) Comparison between media with high versus low vitamin D under conditions without n-3 LCPUFA supplementation. (E) Comparison between media with high versus low vitamin D under conditions with a low concentration of n-3 LCPUFA. (F) Comparison between media with high versus low vitamin D under conditions with a high concentration of n-3 LCPUFA. (G) Comparison between media with low versus no vitamin D under conditions without n-3 LCPUFA supplementation. (H) Comparison between media with low versus no vitamin D under conditions with a low concentration of n-3 LCPUFA. (I) Comparison between media with low versus no vitamin D under conditions with a high concentration of n-3 LCPUFA. Table S8. Replicated differentially expressed genes identified in both cell lines when comparing high-concentration versus no-concentration n-3 LCPUFA treatments. The table lists Ensembl gene IDs, gene symbols, log2 fold changes, standard errors (lfcSE), raw *p*-values, and Benjamini–Hochberg (BH) adjusted *p*-values for each gene in both BILX and SEHP cell lines. Table S9. RNA quality control (QC) metrics for each sample included in the transcriptomic analysis. The table lists sample identifiers, plate and well positions, supplier names, RNA concentrations, volumes, total RNA yield, RNA integrity number (RIN) values, and comments on sample quality.

## Abbreviations

The following abbreviations are used in this manuscript:

n-3 LCPUFA	n-3 long chain polyunsaturated fatty acids
iPSC	Induced pluripotent stem cell
PCoA	Principal coordinates analysis
LC-MS	Liquid chromatography mass spectrometry
PCA	Principal component analysis
qPCR	Quantitative polymerase chain reaction
