# Supplementary material for: Effects of n-3 Long-Chain Polyunsaturated Fatty Acid and Vitamin D Supplementation on Transcriptional Profiles of Human Lung Organoids
Source: Metabolites. 2025 Oct 14;15(10):670. doi: 10.3390/metabo15100670 (PMC12566473; doi:10.3390/metabo15100670)
Supplement: Supplementary file 1 [file metabolites-15-00670-s001.zip › Lung Organoid SUPPLEMENT FIGURES.pdf]

Supplementary Figures

Supplementary Figure S1

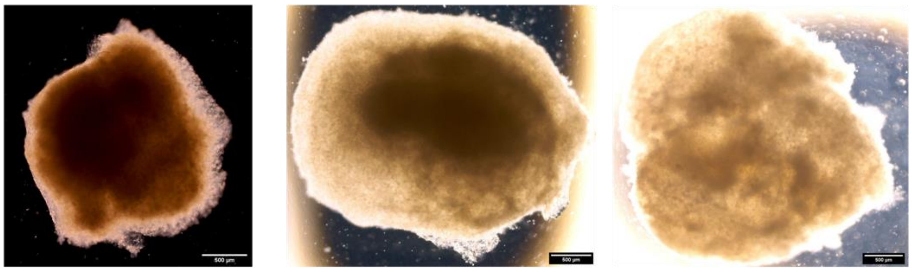

Figure S1: Quality assessment of lung organoid cell cultures in different stages.

Supplementary Figure S2

A)

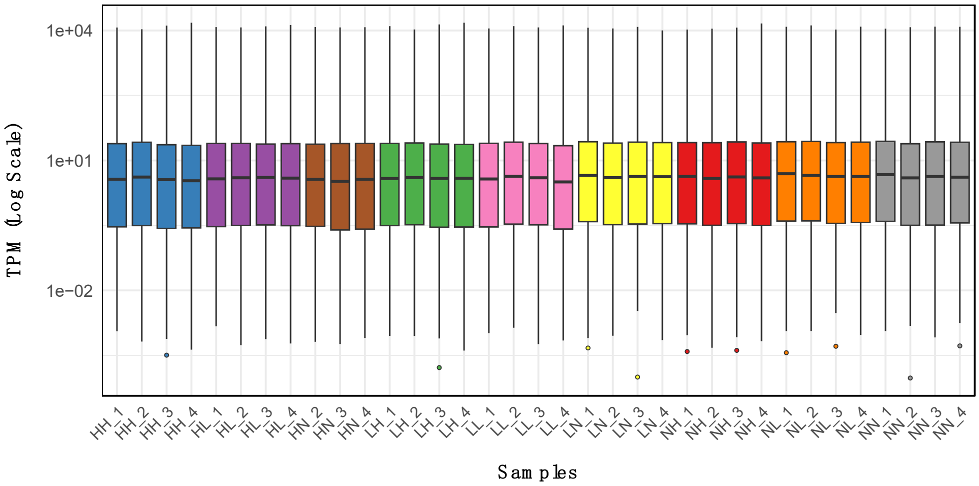

B)

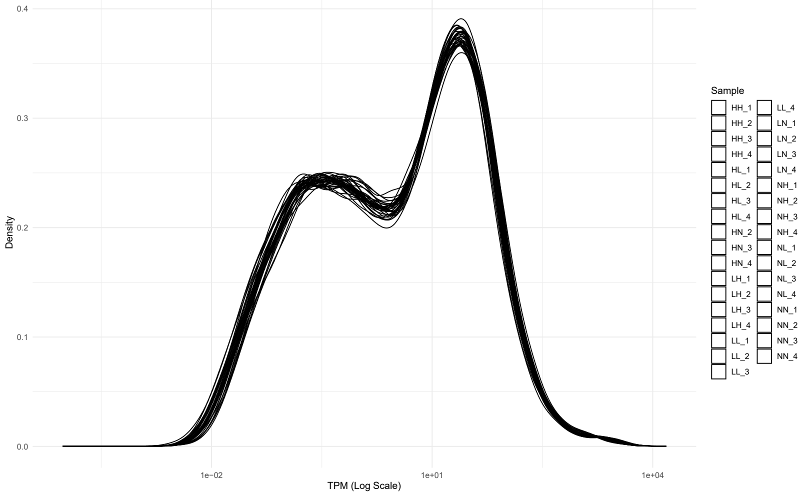

C)

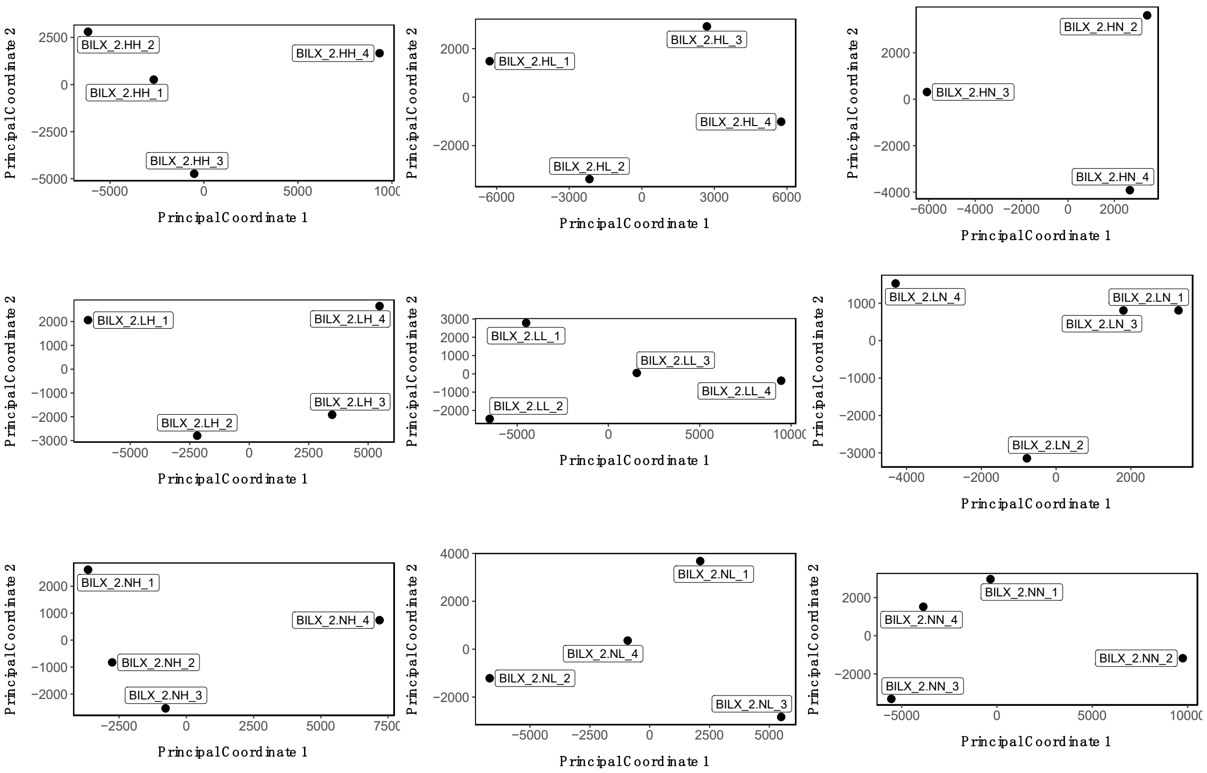

Figure S2: Quality assessment of transcriptome data in the BILX_2 cell line. (A) Boxplots of TPM (Transcripts Per Million) normalized read counts on a log scale. The first character represents the n-3 LCPUFA concentration, and the second character represents the vitamin D concentration, with concentrations designated as follows: N for none, L for low, and H for high. (B) Density plot of TPM normalized read counts. (C) Principal Coordinates Analysis (PCoA) plot of replicates in media with different concentrations of n-3 LCPUFA and vitamin D. The cell line name (BILX_2) is followed by two characters. The first character represents the n-3 LCPUFA concentration, and the second character represents the vitamin D concentration, with concentrations designated as follows: N for none, L for low, and H for high.

Supplementary Figure S3

A)

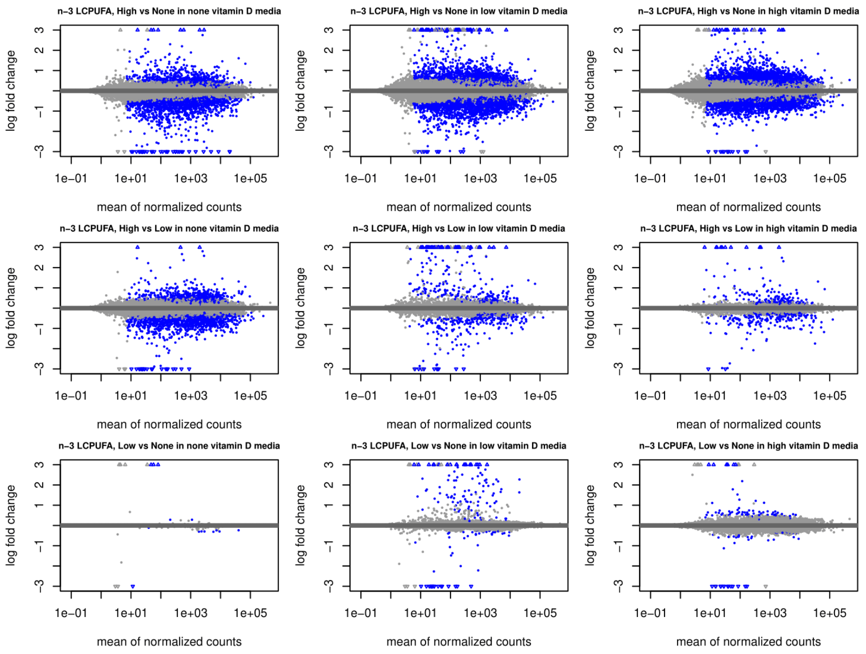

B)

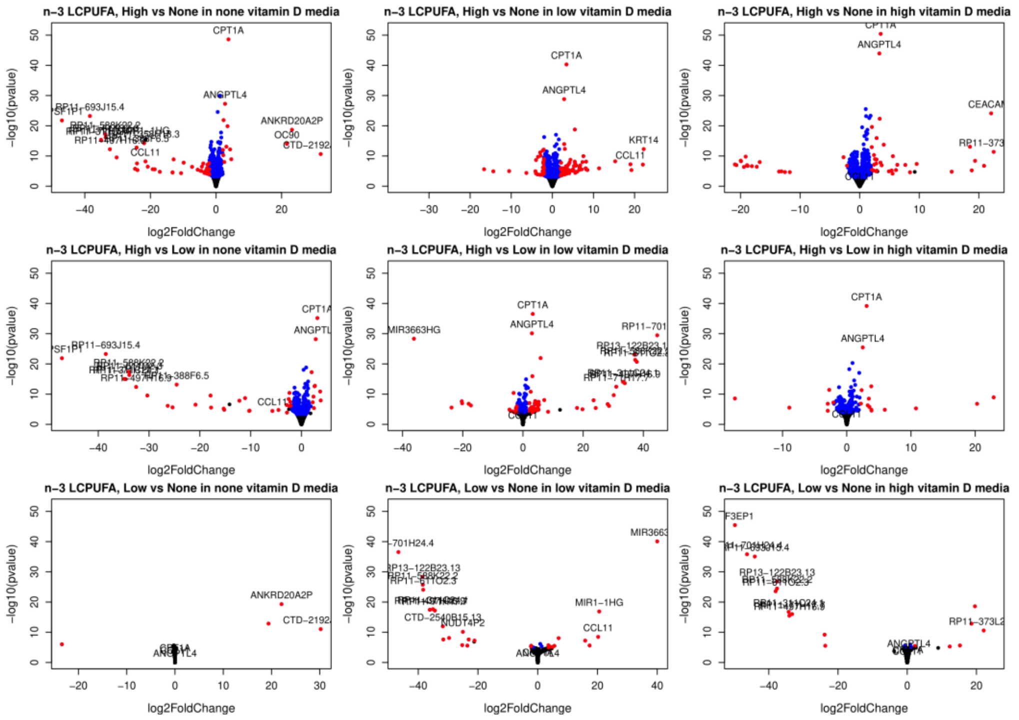

Figure S3: Differential expression analysis between different concentrations of n-3 LCPUFA in media with varying concentrations of vitamin D. **(A)** MA plot of shrunken Log2 fold changes over the mean of normalized counts. Blue points indicate BH adjusted p-value < 0.05. Blue triangles (up/down) mark genes with log2 fold change > 3. **(B)** Volcano plot showing statistical significance and magnitude of change for genes. X-axis shows log2 fold change; Y-axis shows statistical significance (-log10 p-value). Blue points: BH adjusted p-value < 0.01; red points: log2 fold change > 1 and BH adjusted p-value < 0.01. Gene labels are shown for a subset of the genes.

Supplementary Figure S4

A)

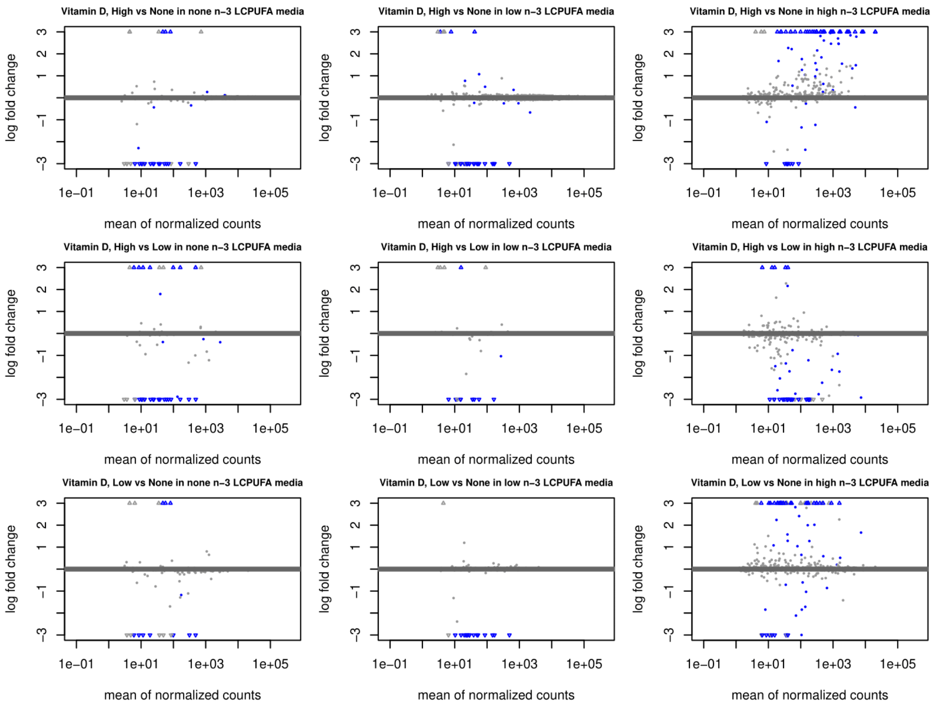

B)

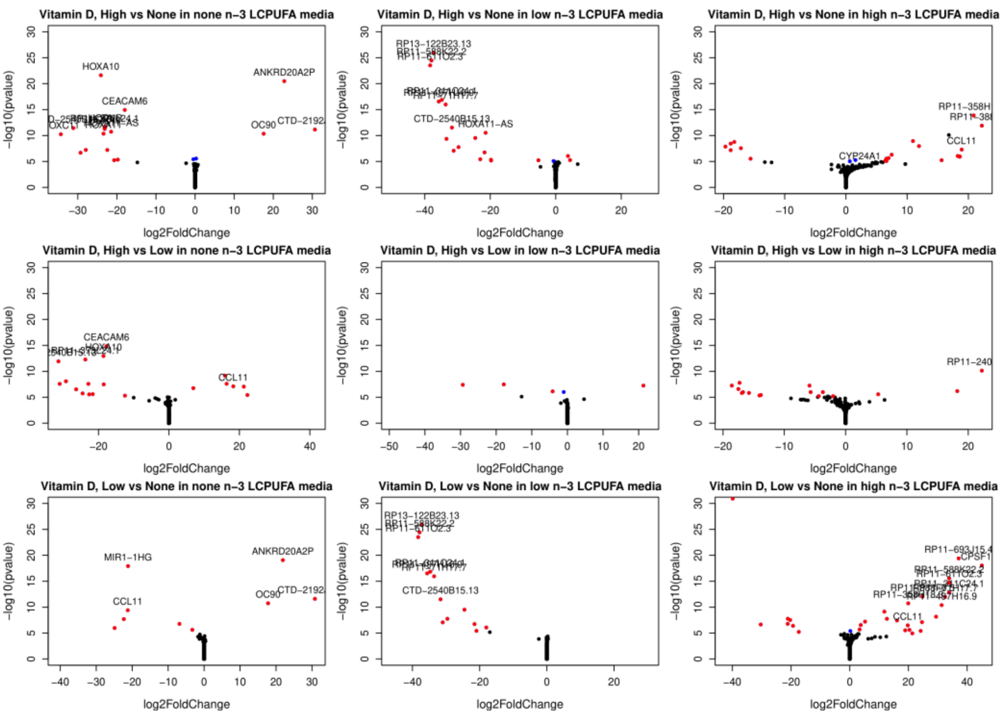

Figure S4: Differential expression analysis between different concentrations of vitamin D in media with varying concentrations of n-3 LCPUFA. **(A)** MA plot of shrunken Log2 fold changes over the mean of normalized counts. Blue points indicate BH adjusted p-value < 0.05. Blue triangles (up/down) mark genes with log2 fold change > 3. **(B)** Volcano plot showing statistical significance and magnitude of change for genes. X-axis shows log2 fold change; Y-axis shows statistical significance (-log10 p-value). Blue points: BH adjusted p-value < 0.01; red points: log2 fold change > 1 and BH adjusted p-value < 0.01. Gene labels are shown for a subset of the genes.

Supplementary Figure S5

A)

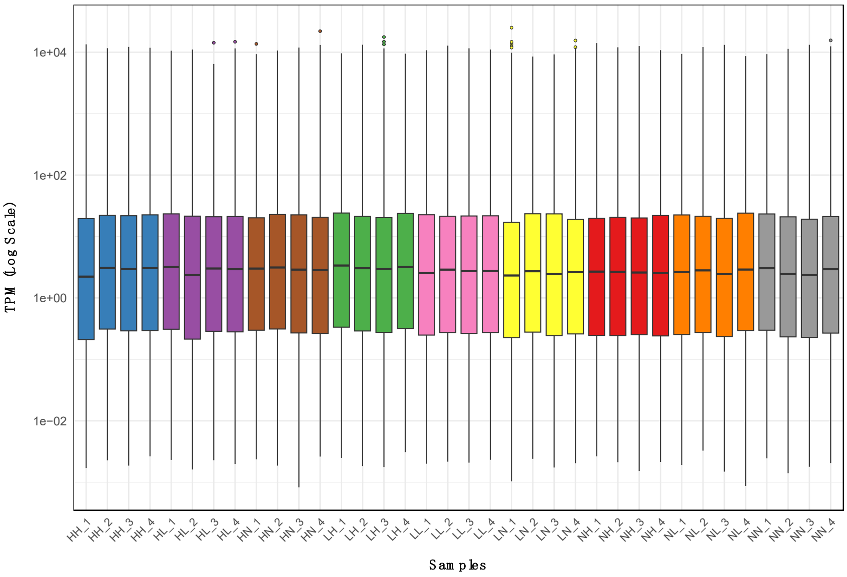

B)

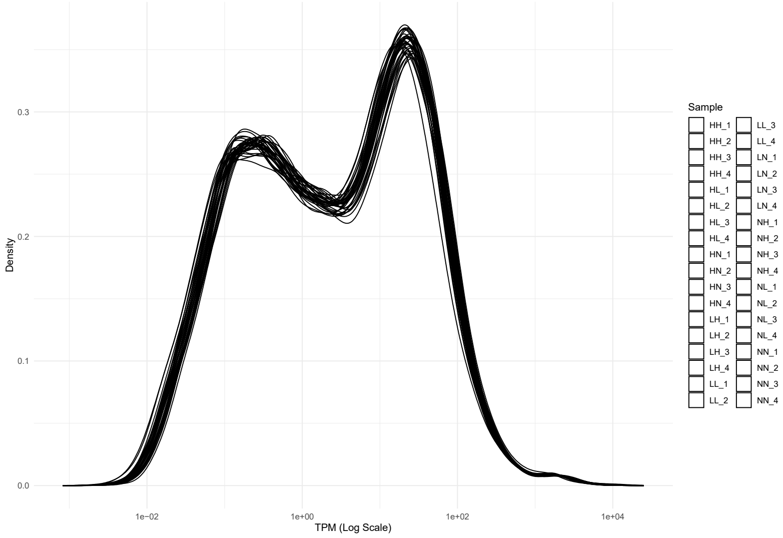

C)

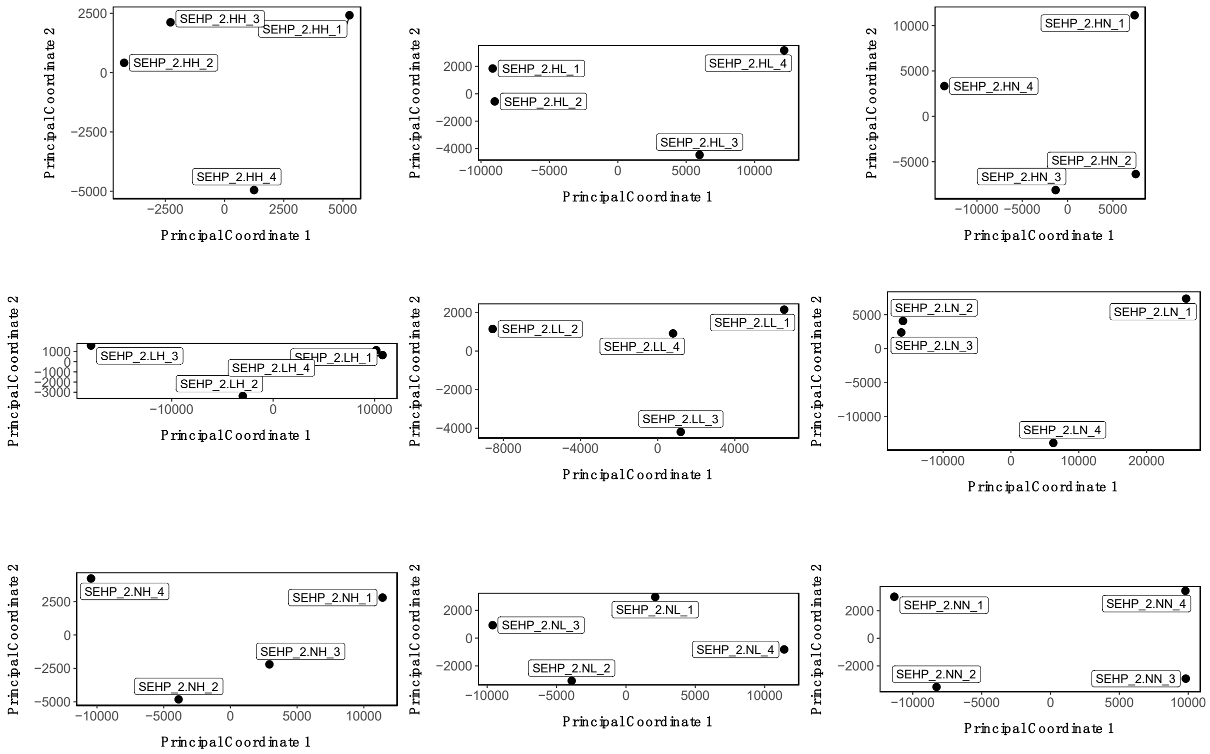

D)

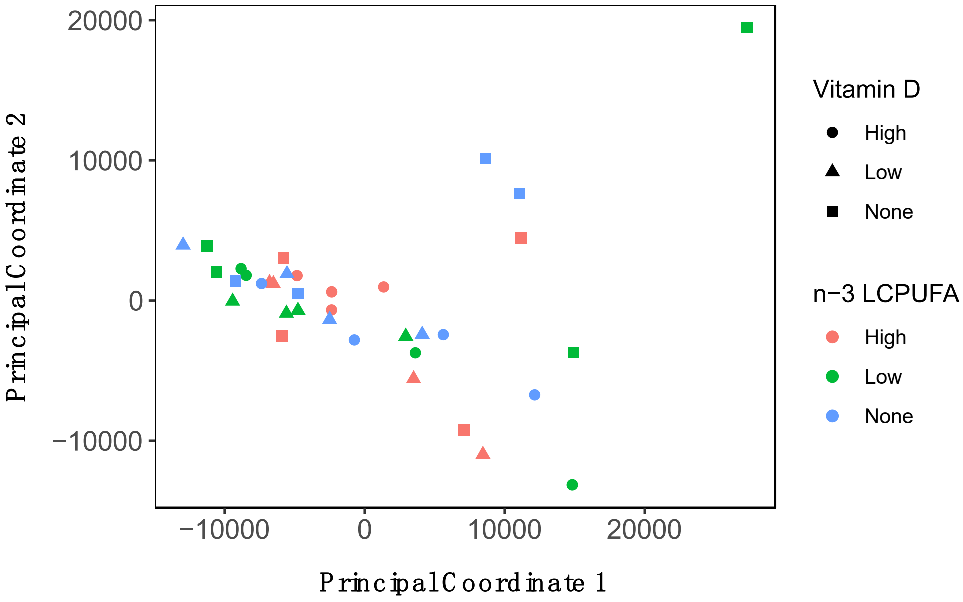

Figure S5: Quality assessment of transcriptome data in the SEHP_2 cell line. (A) Boxplots of TPM (Transcripts Per Million) normalized read counts on a log scale. (B) Density plot of TPM normalized read counts. (C) Principal Coordinates Analysis (PCoA) plot of replicates in media with different concentrations of n-3 LCPUFA and vitamin D. The cell line name (SEHP_2) is followed by two characters. The first character represents the n-3 LCPUFA concentration, and the second character represents the vitamin D concentration, with doses designated as follows: N for none, L for low, and H for high. (D) PCoA plot of replicates: Each dot represents a sample, with dot color indicating the concentration of n-3 LCPUFA and dot shape indicating the concentration of Vitamin D.

Supplementary Figure S6

A)

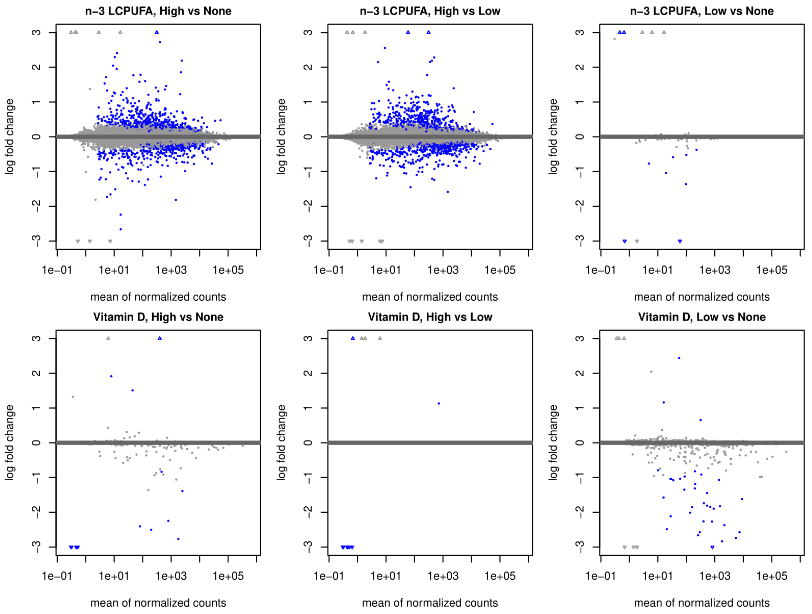

B)

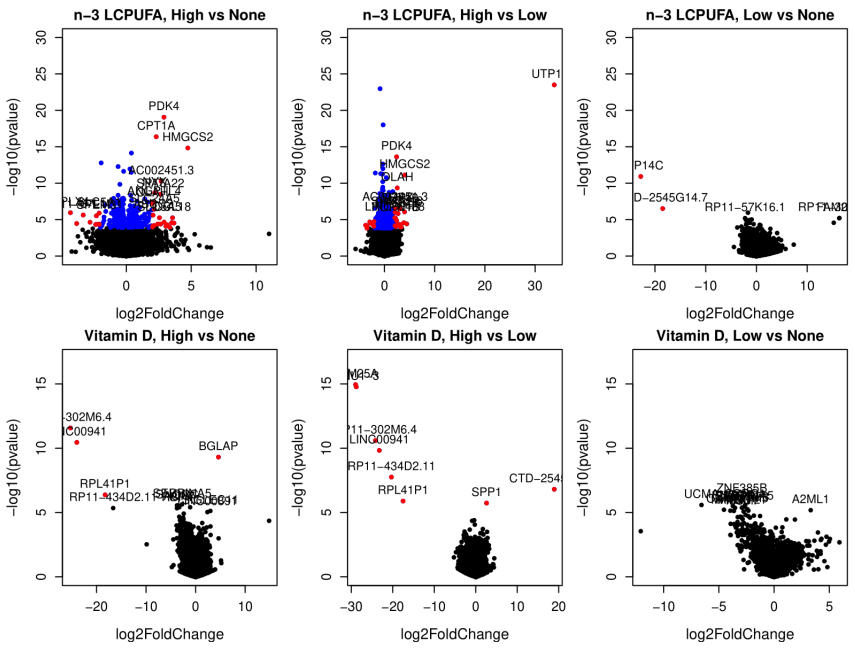

Figure S6: Differential expression analysis between different concentrations of n-3 LCPUFA and vitamin D in the SEHP_2 cell line. **(A)** MA plot of shrunken Log2 fold changes over mean of normalized counts: Points are colored blue if the Benjamini-Hochberg (BH) adjusted p-value is less than 0.05. Points with a log2 fold change greater than 3 are represented as open triangles pointing up or down, indicating substantial expression changes. **(B)** Volcano plot showing statistical significance and magnitude of change for genes. X-axis shows log2 fold change; Y-axis shows statistical significance (-log10 p-value). Blue points: BH adjusted p-value < 0.01; red points: log2 fold change > 1 and BH adjusted p-value < 0.01. Gene labels are shown for a subset of the genes.

Supplementary Figure S7

A)

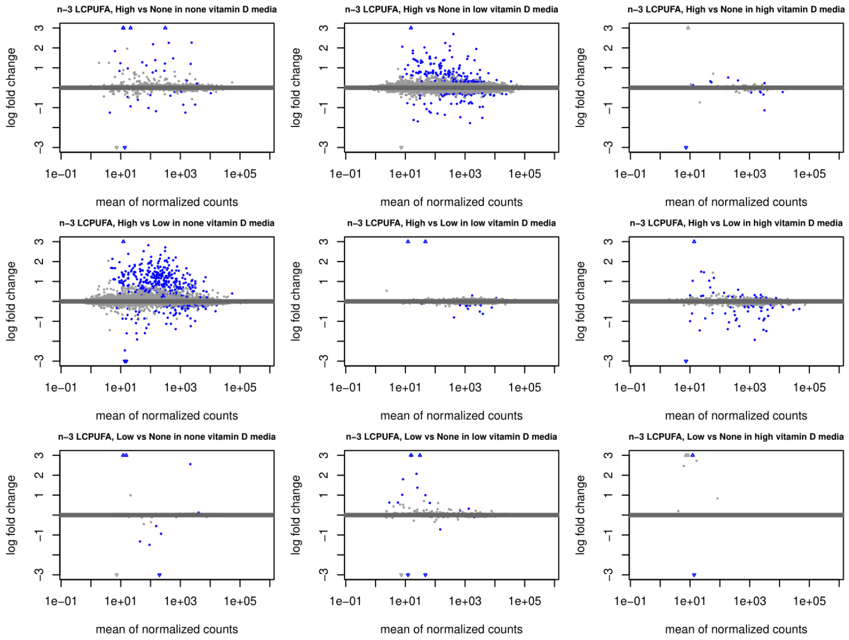

B)

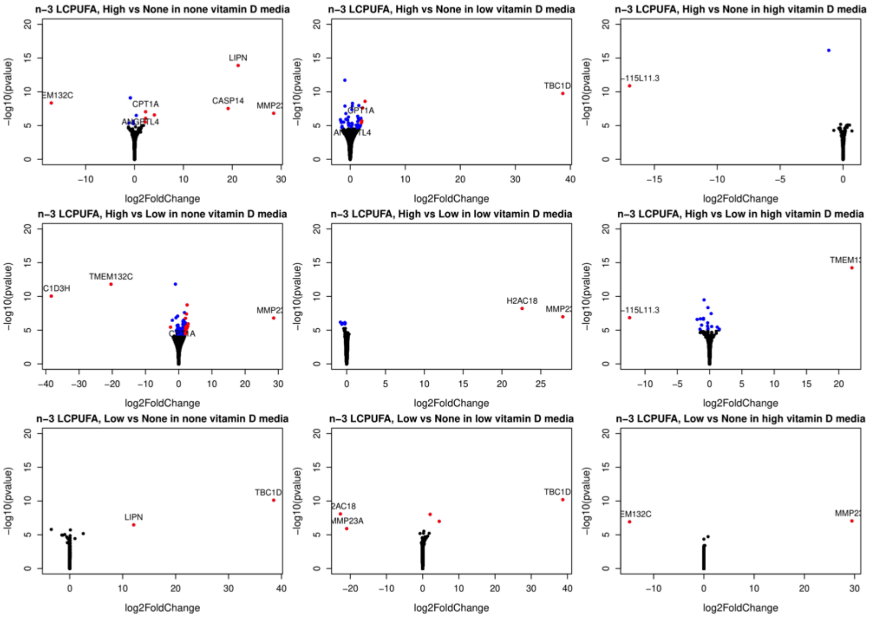

Figure S7: Differential expression analysis between different concentrations of n-3 LCPUFA in media with varying concentrations of vitamin D in the SEHP_2 cell line. **(A)** MA plot of shrunken Log2 fold changes over the mean of normalized counts. Blue points indicate BH adjusted p-value < 0.05. Blue triangles (up/down) mark genes with log2 fold change > 3. **(B)** Volcano plot showing statistical significance and magnitude of change for genes. X-axis shows log2 fold change; Y-axis shows statistical significance (-log10 p-value). Blue points: BH adjusted p-value < 0.01; red points: log2 fold change > 1 and BH adjusted p-value < 0.01. Gene labels are shown for a subset of the genes.

Supplementary Figure S8

A)

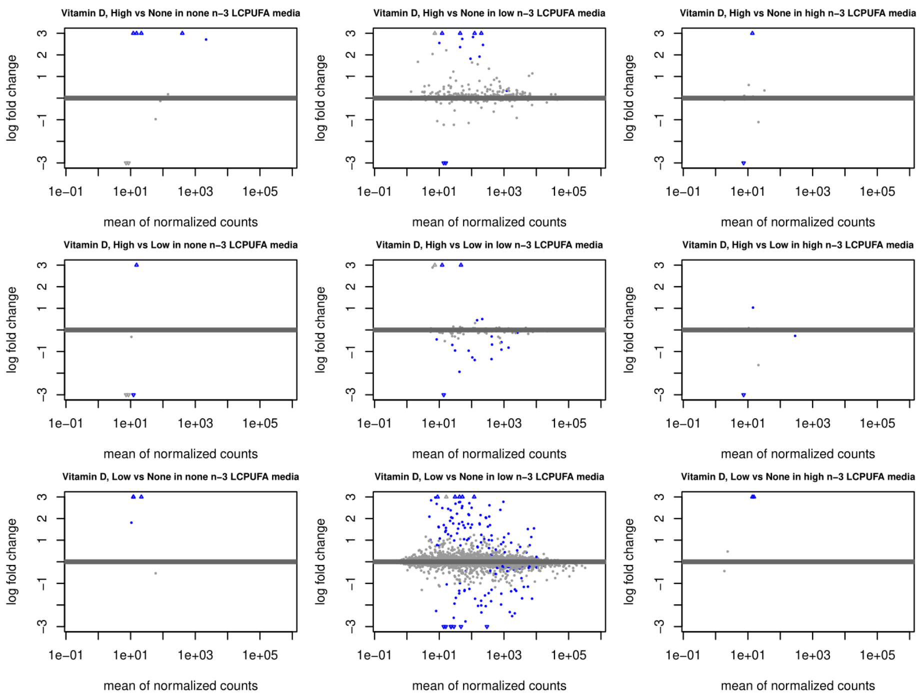

B)

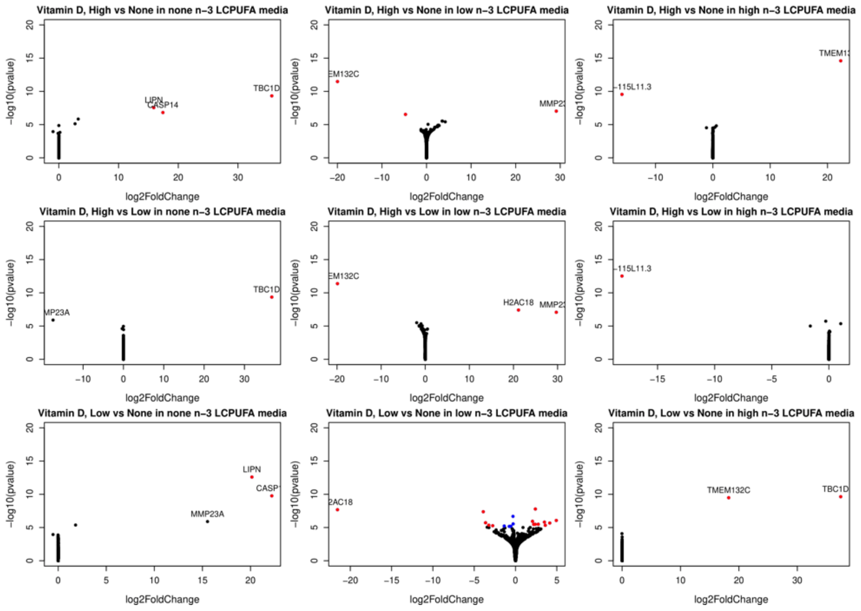

Figure S8: Differential expression analysis between different concentrations of vitamin D in media with varying concentrations of n-3 LCPUFA in the SEHP_2 cell line. **(A)** MA plot of shrunken Log2 fold changes over the mean of normalized counts. Blue points indicate BH adjusted p-value < 0.05. Blue triangles (up/down) mark genes with log2 fold change > 3. **(B)** Volcano plot showing statistical significance and magnitude of change for genes. X-axis shows log2 fold change; Y-axis shows statistical significance (-log10 p-value). Blue points: BH adjusted p-value < 0.01; red points: log2 fold change > 1 and BH adjusted p-value < 0.01. Gene labels are shown for a subset of the genes.

Supplementary Tables

All the supplementary tables are attached in Excel format.
